# Patient Perception of Informed Consent and Its Associated Factors among Surgical Patients Attending Public Hospitals in Dessie City Administration, Northeast Ethiopia

**DOI:** 10.1155/2022/6269921

**Published:** 2022-07-01

**Authors:** Hana Gebrehiwot, Nathan Estifanos, Yosef Zenebe, Tamrat Anbesaw

**Affiliations:** ^1^Wollo University, College of Medicine and Health Sciences, Department of Adult Health Nursing, Dessie, Ethiopia; ^2^Wollo University, College of Medicine and Health Sciences, Department of Comprehensive Nursing, Dessie, Ethiopia; ^3^Wollo University, College of Medicine and Health Sciences, Department of Psychiatry, Dessie, Ethiopia

## Abstract

**Background:**

Poor perception of informed consent compromises patients' autonomy and self-determination; as a result, they feel powerless and unaccountable for their treatment. This study aimed to assess patients' perception of informed consent and its associated factors among surgical patients attending public hospitals in Dessie City Administration, Northeast Ethiopia.

**Methods:**

Facility-based cross-sectional study was conducted on 422 surgical patients. A systematic sampling technique was used to select the study participants. Data were collected using a pretested structured interviewer-administered questionnaire. EpiData version 3.1 was used for data entry, and then data were exported to SPSS version 25 for analysis. Multivariable logistic regression analysis was done to identify factors associated with the outcome variable among the participants. Variables with *p* value less than 0.05 were considered statistically significant factors.

**Results:**

The prevalence of poor perception of informed consent for surgical procedures was found to be 33.2% (95% CI: 28.8–37.8). In multivariable analysis, educational status with inability to read and write (AOR = 5.71; 95% CI: 2.76–11.80) and basic ability to read and write (AOR = 6.03; 95% CI: 2.57–14.16), rural residence (AOR = 3.71; 95% CI: 1.94–7.07), marital status being widowed and divorced (AOR = 3.85; 95% CI: 1.83–8.08), language of written informed consent different from mother tongue (AOR = 4.196; 95% CI: 1.12–15.78), poor patient-physician relationship (AOR = 2.35; 95% CI: 1.31–4.24), and poor knowledge of surgical informed consent (AOR = 3.05; 95% CI: 1.56–5.97) were significantly associated with poor perception of surgical informed consent.

**Conclusion:**

In this study, one-third of surgical patients appear to have poor perceptions of informed consent for surgical procedures. Educational status, being rural residents, being widowed/divorced, language of written informed consent, poor patient-physician relationship, and poor knowledge of surgical informed consent were variables that are independent predictors of poor perception of informed consent for surgical procedures. The ministry of health and healthcare providers should develop a plan to raise patients' awareness about the informed consent process for surgical procedures.

## 1. Introduction

Informed consent is the process by which a patient learns about and understands the purpose, benefits, and potential risks of medical or surgical intervention, including clinical trials, and then agrees to receive the treatment or participate in the trial [1]. Surgical informed consent (SIC) can assist the patients in protecting themselves from unwanted interventions, keep patients' autonomy, and legal/ethical rights. Informed consent, which is a medicolegal requirement, is an important contributing factor to patient satisfaction; in fact, that patient satisfaction is very important for any medical practitioner [2, 3].

The informed consent form (ICF) is a document that should explain the nature and effect of the act and be given to the patients before surgery so that they can decide to undergo the procedure or choose another option [4]. Informed consent eventually emerged as a legal requirement and a right in 1972. This was a result of a series of legal cases in California in 1950 [5]. According to Ethiopian guidelines, Medical Ethics for Doctors in Ethiopia recommends that the doctor has to inform the patient about the treatment (including surgical procedures) she/he intends to carry out. In the process of obtaining informed consent, the doctor is always obliged to obtain the written consent of the patient before carrying out surgeries [6].

Globally, based on the Lancet 2015 report, 313 million surgical procedures are undertaken worldwide each year [7]. In Ethiopia, according to the ministry of health report in 2017, nearly 200,000 operations are performed per year [8]. As the field of global surgery grows to meet the need, ethical considerations need to be addressed [9]. There are universal guidelines on informed consent that are intended to protect patients and promote good ethical practice. Individuals should understand the purpose of the procedure and its process, risks, benefits, and alternatives before giving consent. They should make a free and voluntary decision about the intervention including refusal of treatment [10].

Patients' perception of surgical informed consent includes the perception of the legal status of consent, perception of the scope of consent, perception of importance, and function of the consent form [2, 11, 12]. The poor perception of SIC compromises shared decision-making between the physician and the patient and inhibits patients' self-determination/autonomy, which potentially causes patients to feel powerless, lacking in control, and unaccountable for their treatment, generally resulting in postoperative patient dissatisfaction [12]. Different studies showed that patients believed that they sign the consent because they thought the surgery would not be performed without signing and thought that they would destroy their relationship with their physicians if they said no to the doctors' choice [11, 13, 14]. Literature suggests that every surgical patient should be informed about the intended procedure by the surgeon prior to signing a consent form, but still patients are unaware of this, and in reality this does not happen in clinical settings, particularly in developing nations.

Research done in Saudi Arabia showed that 23.7% of the patients had reported poor perception of surgical informed consent [15]. In addition, another cross-sectional study in Rwanda found that 23% of patients reported poor perception of surgical informed consent [16]. In a study from Ethiopia, Gondar University Hospital showed that 51.3% of patients had a poor perception of surgical informed consent [17]. Several factors are associated with and increase the risk of the development of poor perception, including age, being unmarried, living in a rural area, low levels of education status, poor knowledge of surgical procedures, language of written informed consent, and poor patient-physician relations [16–19].

There are very limited studies conducted in Ethiopia, particularly in the University of Gondar Hospital [17] and Southern Ethiopia [20], but no study was conducted to determine the level of perception of surgical informed consent in surgical patients in Northeast Ethiopia. Therefore, the present study has assessed patients' perception of informed consent and its associated factors among surgical patients attending public hospitals of Dessie City Administration, Northeast Ethiopia. The finding of the study will be important information to the city health department; health bureaus; health professionals who are working with surgical patients in operation rooms, orthopedic wards, surgical wards, and obstetrics/gynecology wards; and other concerned bodies. Additionally, it will serve as a benchmark for other researchers for further investigation in the study areas and similar settings.

## 2. Methods

### 2.1. Study Area and Period

This study was conducted in Dessie City Administration located in South Wollo Zone, Amhara National Regional State, northeast part of Ethiopia, 401 km far from the capital city of Ethiopia, Addis Ababa. Dessie city has 26 kebeles (the lowest administrative level in Ethiopia) (18 urban and 8 rural). According to the 2020 population projection, the total population of Dessie was 385,850. There is one governmental comprehensive specialized hospital and one governmental general hospital in Dessie City Administration. This study was conducted from May 10 to June 15, 2021.

### 2.2. Study Design

An institution-based cross-sectional study was employed.

### 2.3. Population

#### 2.3.1. Source Population

Source population consisted of all surgical patients attending services in public hospitals of Dessie City Administration.

### 2.4. Study Population

Study population consisted of all selected surgical patients attending services in public hospitals of Dessie City Administration during the data collection period.

### 2.5. Eligibility Criteria

#### 2.5.1. Inclusion Criteria

Patients aged ≥18 years and attending services in public hospitals of Dessie City Administration during the data collection period were included in the study.

### 2.6. Exclusion Criteria

Patients who were critically ill, unable to speak and hear, were excluded.

### 2.7. Sample Size Determination and Sampling Procedure

#### 2.7.1. Sample Size Determination

To determine the sample size for perception of informed consent, the single population proportion formula was used by considering the following assumptions: a 5% degree of freedom; 95% confidence interval at alpha (*α*) = 0.05; and population proportion of 51.3%, taken from another study [17]. After adding a 10% nonresponse rate, the final size was 422.

### 2.8. Sampling Technique and Procedure

**Figure 1:**
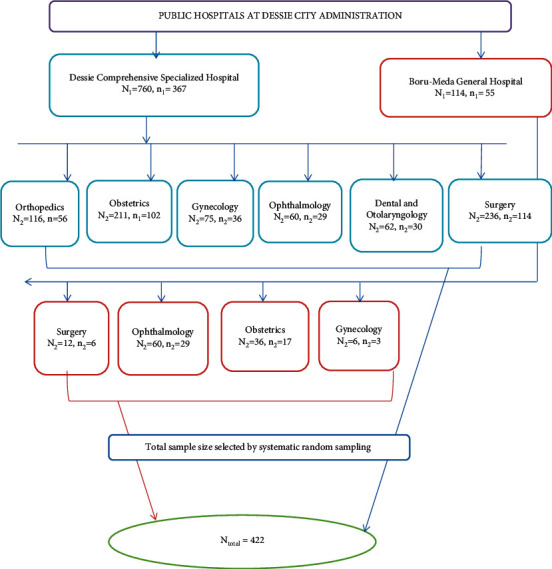
Schematic representation of sampling technique showing the number of selected samples from each public hospital at Dessie City Administration, 2021.

For each of the two hospitals, the sample size was allocated using probability proportionate to the average monthly major surgical client flow (N1) from the registration book in the actual data collection period to make it representative. This was *n*_1_ = 367 for Dessie Comprehensive Specialized Hospital and *n*_1_ = 55 for Boru Meda General Hospital. Then, based on strata that divide surgical patients into different according to the surgical specialties (general surgery, orthopedics, obstetrics, gynecology, ophthalmology, otolaryngology, and dental surgery), we proportionally allocate to each stratum based on each number of surgical patients. The study unit (surgical patient) was selected using systematic sampling by determining the sampling interval (K). By considering K as 2 for both hospitals, the participant was selected every two intervals for one-month and one-week data collection period. Study participants were interviewed daily on working days and weekends during the study period based on their sequence of operations done for each ward, where *N*1 = monthly average number of overall patients with major surgeries for each hospital (Figure 1).

### 2.9. Data Collection Tools and Techniques

An interviewer-administered questionnaire was used. Sociodemographic, clinical-related, and services-related questionnaires were developed by reviewing different kinds of literature [2, 17]. The level of patient perception of informed consent was rated on a Likert scale whereby a higher score indicates a more favorable answer and a lower score indicates a less favorable one, and the overall score was calculated. The minimum possible total score for level of perception was 11, and the maximum possible score was 33. Dividing the score attained in this section by the maximum possible attainable score (33) and multiplying the result by a hundred came up with a percentage calculated level of perception of informed consent for surgical procedures [16]. The validity of the instrument was determined in terms of face, content, and construct validity. These questionnaires were tested in different pilot studies to assess the validity of the questionnaire in studies done in Leicester, England, and Egypt [12, 21]. The presence of perception of surgical informed consent among patients is explained as follows: good perception towards informed consent if a perception of informed consent is above and equal to 70%, whereas poor perception towards informed consent if a perception of informed consent is below 70% [16]. Five questions were selected to assess the level of knowledge towards surgical informed consent, and points were assigned to their answers as follows: 1 if the reply is “Yes” and 0 if it is “No.” The points were totaled, and a score of five was assigned. A patient's knowledge was assessed to be poor if he or she scored 0 to 1, unsatisfactory if he or she scored 2 to 3, satisfactory if he or she scored 4 to 5, and good if he or she scored 4 to 5 [22]. In this study, the overall knowledge was recategorized as follows: a score of 4 to 5 was judged as good knowledge; a score below 4 was categorized as poor knowledge.

The patient-to-provider relationship can be seen as the perception of the patient concerning the caring shown by the provider and the attitude and behavior of the provider toward the patient. In this study, the patient-physician relationship was measured based on the mean score of participants. Responses to the five questions were scored and added. The patient-physician relationship ranged from 0 to 20 [2]. Patients with a mean score of 14.1 or lower had a poor patient-physician relationship, while those with a score of 14.1 or greater had a good patient-to-physician relationship.

### 2.10. Data Quality Control

The questionnaire was initially developed in the English language and then translated into the local language (Amharic) and translated back to English to ensure consistency. To assure the data quality, training was given for seven data collectors (graduate B.S. nurses) and supervisors (two MSc in adult health nursing) for two days by the principal investigator on the objective and the relevance of the study, confidentiality of information, respondents' rights, pretest, informed consent, and techniques of the interview before the data collection. Pretest was done by taking 5% of the total sample size at Hidar 11 Hospital before the actual data collection period to assess instrument simplicity, flow, and consistency, and modifications were done accordingly. Close supervision was done regularly by the supervisors and the principal investigator (PI). They checked and reviewed the questionnaires on daily basis to ensure completeness and consistency of the information. The collected data were edited and entered into the computer from a paper, then checked twice, and processed timely.

### 2.11. Data Processing and Analysis

The collected data was entered into EpiData version 3.1 and exported to SPSS version 26 for analysis. Different frequency tables, graphs, and descriptive summaries were used to describe the study variables. Bivariate and multivariable logistic regression were done to identify the statistically significant association between the covariate and outcome variables. Variables with a *p* value <0.2 in the bivariable analysis were retained for inclusion into the multivariable analysis in the final model to see the relative effect of confounding. In the multivariable analysis, the odds ratio with 95% CI was used to determine the strength of association. Hosmer–Lemeshow test was used to assess the fitness assumption. Variables with *p* value <0.05 were taken as having statistically significant associations.

## 3. Results

### 3.1. Sociodemographic Characteristics of the Participants

A total of 422 postsurgical patients were involved in this study with a 100% overall response rate. The majority of participants (163, 38.6%) ranged from 18 to 30 years old. Nearly two-thirds (263, 62.3%) of the respondents were female, and almost two-thirds (290, 68.7%) were married. Almost all (387, 91.7%) were Amhara in ethnicity. 165 (39.1%) of the participants were Orthodox Christian followers. Regarding the educational status, 136 (32.2%) of them were unable to read and write. 172 (40.8%) were farmers by occupation, and more than half (218, 51.7%) were living in the urban area (Table 1).

### 3.2. Clinical-Related Characteristics of the Participants

According to this study finding, the majority (120, 28.5%) of patients had general surgery. The study revealed that more than half (232, 55.0%) had an elective schedule of surgery. Nearly one-fourth (102, 24.2%) of the respondents had a history of chronic medical illness, the majority of which (43, 42.2%) reported a history of hypertension. Nearly one-fourth of the participants (94, 22.3%) had a previous surgical history, most of which (76, 80.9%) had a one-time history of surgery (Table 2).

### 3.3. Service-Related Characteristics of the Participants

In this study finding, 407 (96.4%) of participants reported that the informed consent was written in their mother tongue. More than half (238, 56.4%) of the respondents were requested to sign the consent form by a nurse. Two hundred thirty-one (54.7%) of the respondents gave consent just prior to the operation. The majority of the respondents (376, 89.1%) signed informed consent by themselves. Almost all (406, 96.2%) of participants took <5 minutes to provide informed consent (Table 3).

### 3.4. Patient-Physician Relationship

The mean score (standard deviation (SD)) of the patient-physician relationship was 14.1 (±4.1). Two hundred twenty six (53.6%) of the respondents allocated a low score to their relationship with their physicians, whereas the remaining, 46.4%, gave a high score for their relationship with their physicians (Table 4).

### 3.5. Knowledge and Understanding of Surgical Informed Consent

91 (21.6%) of the respondents signed informed consent previously. Overall, 198 (46.9%) respondents had good levels of knowledge about SIC (Table 5).

### 3.6. Patient Perception of Informed Consent for Surgical Procedures

In this study, 140 (33.2%, 95% CI: 28.8–37.8) had poor perceptions of informed consent for surgical procedures. The rest, 66.8% (95% CI: 62.2–71.2), had a good perception of informed consent for surgical procedures.

### 3.7. Factors Associated with Poor Perception of Informed Consent for Surgical Procedures

In the bivariate analysis, the variables age, educational status, living in a rural area, being widowed/divorced, having an elective schedule of surgery, being obstetric and ophthalmology patients, history of chronic medical illness, having a poor patient-physician relationship, and having poor knowledge about surgical informed consent showed a *p* value of <0.20 and became candidates for multivariable analysis. In multivariable binary logistic regression, the variables educational status, being widowed/divorced, being rural residents, language of written informed consent different from the mother tongue, having a poor patient-physician relationship, and having poor knowledge of surgical informed consent were found to be statistically associated with poor perceptions of surgical informed consent at a *p* value less than 0.05.

Surgical patients with inability to read and write were 5.71 times (AOR = 5.71; 95% CI: 2.76–11.80) and those with basic ability to read and write were 6 times (AOR = 6.03; 95% CI: 2.57–14.16) more likely to have poor perception of surgical informed consent compared to those with formal educational status. Rural residents were 3.71 times more likely to have a poor perception of surgical informed consent as compared to urban residents (AOR = 3.71; 95% CI: 1.94–7.07). The odds of having poor perception of surgical informed consent with widowed/divorced patients were nearly four times higher as compared to married ones (AOR = 3.85; 95% CI: 1.83–8.08). Likewise, patients having informed consent written in a language different from their mother tongue were four times more likely to have a poor perception of surgical informed consent than those having IC written in their mother tongue (AOR = 4.196; 95% CI: 1.12–15.78).

Furthermore, those surgical patients who had poor patient-physician relationships were two times more likely to have a poor perception of surgical informed consent than those who had good patient-physician relationships (AOR = 2.35; 95% CI: 1.31–4.24). Finally, surgical patients who had poor knowledge of surgical informed consent were three times more likely to have a poor perception of surgical informed consent than those who had good knowledge of surgical informed consent (AOR = 3.05; 95% CI: 1.56–5.97) (Table 6).

## 4. Discussion

In this study, 33.2% (95% CI: 28.8–37.8) of the patients had a poor perception of informed consent for surgical procedures. This percentage is lower than that of a study done at Gondar University Hospital, where 51.3% had a poor perception of surgical informed consent [17]. This discrepancy might be due to variation in used inclusion criteria and demographic characteristics of the populations. However, this finding was a higher result as compared with those in the studies done in Saudi Arabia [15] and Rwanda [16], where 23.7% and 23%, respectively, had a low perception of informed consent for surgical procedures. The difference might be the sample size (147), study setting, and regression models (ordinal logistic) used in Rwanda. In addition, our result was higher than that in the study done in Nigeria, where the majority (97.0%) had a good perception of surgical informed consent [13]. The variation might be due to sampling technique difference as non-probability type of purposive sampling was used in Nigeria. Moreover, unlike our study, most of the study participants (89.4%) were educated, and this might also be another thing that makes perception difference in two study areas. Furthermore, the variation might be due to numerous factors including differences in the characteristics of study participants, sociocultural variation, and inclusion and exclusion criteria used.

Regarding the associated factors, surgical patients with inability to read and write were 5.71 times and those with basic ability to read and write were 6 times more likely to have poor perception of informed consent for surgical procedures compared to those with formal educational status. This finding was in agreement with a study in Gondar University Hospital, Ethiopia [17]; South Africa [3]; and Rwanda [16]. The possible justification might be that patients with lower levels of education status were unable to read the written IC form [23] and did not fully understand the principle and practice of informed consent policy [18]. As a result, they may have a poor perception of informed consent for surgical procedures. Additionally, patients' educational background is related to the level of attention they give to information provided by the physician and their ability to describe this information when asked. For this reason, participants with lower educational status grasped less information as compared to patients with higher education.

In the current study, widowed/divorced patients were nearly four times more likely to have a poor perception of surgical informed consent as compared to married patients. The reason might be a lower level of emotional and perceived social support derived from sharing information and consulting with the partner before making a decision. Inability to share information with partners increases stress, as well as reducing self-esteem and happiness, and in turn leads to poor perception for informed consent [24].

This finding also revealed that rural residents were 3.71 times more likely to have a poor perception of surgical informed consent as compared to urban residents. This was supported by the study conducted in Gondar University Hospital [17], Rwanda [16], and Nigeria [13], which showed that study participants living in rural areas were more likely to have poor perceptions than in those living in urban areas. This might be because most of the time in rural settings there is less access to different sources of information, and less educated populations are living in rural areas.

The current study also showed that patients having informed consent written in a language different from their mother tongue were 4 times more likely to have a poor perception of surgical informed consent than those having IC written in their mother tongue. This is supported by the finding in Southern Ethiopia [20]. The possible explanation might be that there is an impact of language barriers on informed consent being properly obtained prior to procedures. This might imply the fact that the provision of information does not guarantee shared understanding due to the language barrier. Additionally, counseling aids prepared in various languages and consistent availability of multilingual counselors would mitigate poor patient perception of informed consent.

Another predictor for poor perception of informed consent was poor patient-physician relationships. Those patients who had poor patient-physician relationships were two times more likely to have poor perception of surgical informed consent for procedures than those who have good patient-physician relationships. The current study finding was congruent with a finding from Gondar University Hospital [17] and Rwanda [16]. In this case, poor patient-physician relationships, including less respect, less openness/honest communication, and less information delivered to the patient from the physician, promote a less effective IC process [2]. Additionally, patients who do not trust their physicians do not fully understand and are not involved in the treatment plan.

Finally, having poor knowledge of surgical informed consent was significantly associated with a poor perception of informed consent for surgical procedures. This is similar to a study done in Rwanda [16]. Knowledge contributes to increased perception of informed consent for surgical procedures. Health literacy is a problem in developing countries because a great majority are less educated.

### 4.1. Limitations of the Study

Since data were collected by face-to-face interview method, they might be prone to social desirability bias. Patients might have forgotten information given to them during the informed consent process (recall bias) since they were interviewed after the surgery, which was considered as a limitation of the study. However, this study included public institutions, which were one comprehensive specialized hospital and one general hospital, rather than a single institution.

## 5. Conclusions

In this study, one-third of surgical patients had a poor perception of informed consent for surgical procedures. Educational status, rural residence, widowed/divorced marital status, language of written informed consent different from patient mother tongue, poor patient-physician relationship, and poor knowledge about surgical informed consent were associated with poor perception of informed consent for surgical procedures. So, it is suggested that the ministry of health and health professionals should plan strategies to enhance the awareness of patients about surgical informed consent.

## Figures and Tables

**Table 1:** Sociodemographic characteristics of surgical patients in Dessie City Administration public hospitals, Northeast Ethiopia, 2021 (*N* = 422).

Variable	Category	Frequency	Percent (%)
Sex	Male	159	37.7
	Female	263	62.3

Age	18–30 years	163	38.6
	31–40 years	96	22.7
	41–50 years	43	10.2
	>50 years	120	28.4

Marital status	Married	290	68.7
	Single	64	15.2
	Divorce	43	10.2
	Widowed	25	5.9

Ethnicity	Amhara	387	91.7
	Oromo	30	7.1
	Others^*∗*^	5	1.2

Religion	Orthodox	165	39.1
	Muslim	241	57.1
	Protestant	13	3.1
	Catholic	3	0.7

Educational status	Inability to read and write	136	32.2
	Basic ability to read and write	45	10.7
	Primary education (1–8)	85	20.1
	Secondary education (9–12)	69	16.4
	College and above	87	20.6

Occupation	Farmer	172	40.8
	Housewife	80	19.0
	Student	19	4.5
	Governmental employed	69	16.4
	Unemployed	9	2.1
	Soldier	68	16.1
	Others ^*∗∗*^	5	1.2

Residence	Urban	218	51.7
	Rural	204	48.3

Note. ^*∗*^Tigre, Gurage; ^*∗∗*^NGO employee, merchant.

**Table 2:** Clinical-related characteristics of surgical patients in Dessie City Administration public hospitals, Northeast Ethiopia, 2021 (*N* = 422).

Variable	Category	Frequency	Percent (%)
Surgery discipline	Orthopedic	56	13.3
	Obstetric	119	28.2
	Gynecology	39	9.20
	Ophthalmology	58	13.7
	Dental and otolaryngology	30	7.10
	Surgery	120	28.5

Schedule of surgery	Elective	232	55.0
	Emergency	190	45.0

Previous chronic medical history	Yes	102	24.2
	No	320	75.8

Type of chronic medical disease	Diabetes mellitus	30	29.4
	Hypertension	43	42.2
	Heart disease	10	9.8
	Asthma	7	6.9
	Psychiatric disorder	3	2.9
	Others^*∗*^	9	8.8

Previous surgical history	Yes	94	22.3
	No	328	77.7

Number of operations Previously done	One	76	80.9
	Two	15	16.0
	Three	3	3.2

Note. ^*∗*^Arthritis and cancer.

**Table 3:** Service-related characteristics of surgical patients in Dessie City Administration public hospitals, Northeast Ethiopia, 2021 (*N* = 422).

Variable	Category	Frequency	Percent (%)
Language of written informed consent	Mother tongue	407	96.4
	Another language	15	3.6

Healthcare worker who explains surgical informed consent process	Nurse	238	56.4
	Intern	29	6.9
	Surgeon	155	36.7

Time of taking consent	On the day before surgery	46	10.9
	On the day of surgery	144	34.1
	Immediately before surgery	231	54.7
	On the operation table	1	0.2

Signee of informed consent	Self	376	89.1
	Parent/spouse	28	6.6
	Relatives	18	4.3

Time taken to provide informed consent	<5 minutes	406	96.2
	5–10 minutes	15	3.6
	>10 minute	1	0.2

**Table 4:** Patient-physician relationship of surgical patients in Dessie City Administration public hospitals, Northeast Ethiopia, 2021 (*N* = 422).

Variables	Never (0) N (%)	Seldom (1) N (%)	Sometimes (2) N (%)	Often (3) N (%)	Always (4) N (%)
Trusting your surgeon	3 (0.7)	17 (4.0)	51 (12.1)	185 (43.8)	166 (39.3)
Feeling comfortable with your surgeon	18 (4.3)	46 (10.9)	78 (18.5)	147 (34.8)	133 (31.5)
Respecting your surgeon's opinion	2 (0.5)	7 (1.7)	32 (7.6)	235 (55.7)	146 (34.6)
Expressing your concerns about the operation to the surgeon	75 (17.8)	92 (21.8)	103 (24.4)	68 (16.1)	84 (19.9)
Feeling that the surgeon heard and understood your opinions and concerns	20 (4.7)	29 (6.9)	80 (19.0)	163 (38.6)	130 (30.8)

**Table 5:** Surgical patients' knowledge and understanding of informed consent in Dessie City Administration public hospitals, Northeast Ethiopia, 2021 (*N* = 422).

Variables	Category	
	Yes (%)	No (%)
Signing surgical informed consent previously	91 (21.6)	331 (78.4)
Reading the surgical informed consent form	106 (25.1)	316 (74.9)
Knowing the operating surgeon	206 (48.8)	216 (51.2)
Knowing the reason for surgery	386 (91.5)	36 (8.5)
Knowing the type/nature of surgery done	307 (72.7)	115 (27.3)
Knowing the options of alternative treatment	266 (63.0)	156 (37.0)
Knowing the anesthesia risks	201 (47.6)	221 (52.4)
Knowing the risks and complications of surgery	210 (49.8)	212 (50.2)
Knowing the expected time the surgery will take	80 (19.0)	342 (81.0)
Knowing the postoperative care	346 (82.0)	76 (18.0)
Knowing what to eat after surgery	330 (78.2)	92 (21.8)
Knowing when to resume working	170 (40.3)	252 (59.7)
Knowing the cost of treatment	59 (14.0)	363 (86.0)

**Table 6:** Factors associated with perception of informed consent for surgical procedures among surgical patients in Dessie City Administration public hospitals, Northeast Ethiopia, 2021 (*N* = 422).

Variables	Category	Perception		COR (95%CI)	AOR (95%CI)
		Poor	Good		
Age	18–30 years	30	133	0.15 (0.09–0.25)	0.97 (0.35–2.74)
	31–40 years	19	77	0.16 (0.09–0.30)	0.60 (0.23–1.56)
	41–50 years	18	25	0.46 (0.23–0.94)	0.69 (0.27–1.75)
	>50 years	73	47	1	1

Educational status	Inability to read and write	91	45	18.28 (10.52–31.77)	5.71 (2.76–11.8)^*∗*^
	Basic ability to read and write	25	20	11.3 (5.48–23.3)	6.03 (2.57–14.16)^*∗*^
	Formal education	24	217	1	1

Marital status	Married	77	213	1	1
	Single	13	51	0.71 (0.36–1.37)	1.50 (0.63–3.58)
	Widowed/divorced	50	18	7.68 (4.22–13.98)	3.85 (1.83–8.08)^*∗*^

Residence	Rural	118	86	12.2 (7.26–20.58)	3.71 (1.94–7.07)^*∗*^
	Urban	22	196	1	1

Schedule of surgery	Emergency	42	148	0.39 (0.25–0.60)	0.74 (0.38–1.47)
	Elective	98	134	1	1

Surgery discipline	Orthopedic	22	34	1.40 (0.72–2.70)	0.99 (0.40–2.46)
	Obstetric	24	95	0.55 (0.30–0.98)	0.19 (0.47–3.06)
	Gynecology	10	29	0.74 (0.33–1.68)	1.17 (0.38–3.55)
	Ophthalmology	33	25	2.85 (1.49–5.44)	1.53 (0.63–3.74)
	Dental and Otolaryngology	13	17	1.65 (0.73–3.74)	1.43 (0.40–5.17)
	Surgery	38	82	1	1

Previous chronic medical history	Yes	54	48	3.06 (1.93–4.85)	1.34 (0.62–2.87)
	No	86	234	1	1

Previous surgical history	Yes	24	70	0.63 (0.37–1.05)	1.09 (0.46–2.58)
	No	116	212	1	1

The language of written informed consent	Different from mother tongue	8	7	2.38 (0.85–6.71)	4.196 (1.12–15.78)^*∗*^
	Mother tongue	132	275	1	1

Patient-physician relationship	Poor	102	124	3.42 (2.20–5.31)	2.35 (1.31–4.24)^*∗*^
	Good	38	158	1	1

Informed consent knowledge	Poor	119	105	9.55 (5.66–16.12)	3.05 (1.56–5.97)^*∗*^
	Good	21	177	1	1

*Note*. ^*∗*^Statistically significant, COR: crude odds ratio, AOR: adjusted odds ratio, and 1: reference category.

## Data Availability

All raw data the current study can be obtained from the corresponding author upon reasonable request.

## Abbreviations

AOR: Adjusted odds ratio
BMGH: Boru Meda General Hospital
CI: Confidence interval
DCSH: Dessie Comprehensive Specialized Hospital
HCW: Healthcare worker
IC: Informed consent
ICF: Informed consent form
NGO: Nongovernmental organization
SIC: Surgical informed consent
SPSS: Statistical Package for the Social Sciences.
